# Impact of Early Growth on Postprandial Responses in Later Life

**DOI:** 10.1371/journal.pone.0024070

**Published:** 2011-08-31

**Authors:** Mia-Maria Perälä, Liisa M. Valsta, Eero Kajantie, Jaana Leiviskä, Johan G. Eriksson

**Affiliations:** 1 Department of Chronic Disease Prevention, The National Institute for Health and Welfare, Helsinki, Finland; 2 Department of Lifestyle and Participation, The National Institute for Health and Welfare, Helsinki, Finland; 3 Hospital for Children and Adolescents, Helsinki University Central Hospital and University of Helsinki, Helsinki, Finland; 4 Department of General Practice and Primary Health Care, University of Helsinki, Helsinki, Finland; 5 Unit of General Practice, Helsinki University Central Hospital, Helsinki, Finland; 6 Folkhälsan Research Center, Helsinki, Finland; 7 Vasa Central Hospital, Vasa, Finland; Innsbruck Medical University, Austria

## Abstract

**Background:**

Low birth weight and slow growth during infancy are associated with increased rates of chronic diseases in adulthood. Associations with risk factors such as fasting glucose and lipids concentrations are weaker than expected based on associations with disease. This could be explained by differences in postprandial responses, which, however, have been little studied. Our aim was to examine the impact of growth during infancy on postprandial responses to a fast-food meal (FF-meal) and a meal, which followed the macro-nutrient composition of the dietary guidelines (REC-meal).

**Methodology/Principal Findings:**

We recruited 24 overweight 65–75 year-old subjects, 12 with slow growth during infancy (SGI-group) and 12 with normal early growth. All the subjects were born at term. The study meals were isocaloric and both meals were consumed once. Plasma glucose, insulin, triglycerides (TG) and free fatty acids (FFA) were measured in fasting state and over a 4-h period after both meals. Subjects who grew slowly during infancy were also smaller at birth. Fasting glucose, insulin or lipid concentrations did not differ significantly between the groups. The TG responses were higher for the SGI-group both during the FF-meal (*P* = 0.047) and the REC-meal (*P* = 0.058). The insulin responses were significantly higher for the SGI-group after the FF-meal (*P* = 0.036). Glucose and FFA responses did not differ significantly between the groups.

**Conclusions:**

Small birth size and slow early growth predict postprandial TG and insulin responses. Elevated responses might be one explanation why subjects who were small at birth and experiencing slow growth in infancy are at an increased risk of developing cardiovascular diseases in later life.

## Introduction

Epidemiological studies have shown that low birth weight is associated with an increased risk of several chronic diseases in later life, as reviewed by Huxley et al [1] and Whincup et al [2]. The Developmental Origins of Health and Disease (DOHaD) hypothesis suggests that a sub-optimal environment in utero alters growth and metabolism to ensure survival [3]. These adaptations result in long term alterations in the structure and function of various organs and may result in adverse health outcomes in adult life [4]. There is also evidence that sensitive early life periods are not limited to fetal life, for example specific growth patterns reflecting conditions during infancy may increase the probability of unfavorable metabolic outcomes and diseases [5]–[10].

Several studies have reported an association between small size at birth and slow growth during infancy and fasting hyperglycemia, insulin resistance [10] and unfavorable lipid profiles [11]–[14]. Associations between birth size and risk factors of cardiovascular diseases (CVD) such as fasting lipids concentrations [11]–[14] are weaker than expected based on associations between birth size and CVD [1], [5]. Only few studies have examined the effect of birth weight on postprandial responses to mixed meals [15]–[17]. To our knowledge, no previous studies have assessed the effect of growth during infancy on postprandial responses. However, people are spending most of their waking hours in a postprandial state, and it has been shown that elevated postprandial responses are stronger predictors of CVD than fasting levels [18]–[20]. Elevated postprandial responses of glucose and lipids may cause accumulation of atherogenic particles and generate excess free radicals that may have effect on inflammation and endothelial dysfunction [21], [22]. These postprandial changes can lead to the development of CVD when repeating many times daily.Therefore, alterations in postprandial responses could potentially explain in part the associations of early growth with adult diseases that are not explained by conventional risk factors such as fasting concentrations of glucose, insulin and lipids.

The aim of the present study was to examine the impact of growth during infancy on triglycerides (TG), free fatty acids (FFA), glucose, and insulin postprandial responses to a fast-food meal (FF-meal) and a meal, which followed the macronutrient composition of the dietary guidelines (REC-meal).

## Methods

### Ethics statement

This study was conducted according to the guidelines laid down in the Declaration of Helsinki, and all procedures involving human subjects were approved by the Ethics Committee of Hospital District of Helsinki and Uusimaa. Written informed consent was obtained from all the subjects.

### Participants

We recruited 24 overweight or obese (body mass index, BMI, 25–32 kg/m^2^) 65–75 year-old subjects, 12 with slow growth during infancy (SGI-group) and 12 with normal early growth (CON-group) from the Helsinki Birth Cohort Study participants who had attended a clinical study [13] and who were born at term. Data on childhood growth were based on repeated measurements at child welfare clinics which have been described in detail [9]. Growth during infancy was examined as gains in BMI between birth and 1 year. We converted each measurement of BMI for each individual to a Z-score, which represents the difference from the mean value for the whole study group (n = 2003) and is expressed in standard deviations. We examined how much BMI at 1 year of age differed from that predicted by the BMI at birth by using the residual from linear regression; we called this measure “conditional growth”. Subjects with slow early growth had conditional growth <−0.9 SD, and the conditional growth of the control group was >−0.9 SD. The conditional growth cut-off point was based on the previous study of our cohort [13] which showed that a 1 SD higher conditional BMI at 24 months was associated with 3.9% lower serum TG concentration.

Glucose tolerance was assessed by a 75 g 2-h oral glucose tolerance test (OGTT), and diabetics were excluded. Other exclusion criteria were smoking, milk allergy, regular medication that would have an effect on postprandial glucose or lipids metabolism (e.g. antidiabetic drugs, fibrates, glucocorticoids), gastrointestinal disease influencing absorption, or a first-degree family history of diabetes. In addition, we excluded those who were born pre-term (before 37 weeks of gestation). Diet, health and lifestyle data were assessed by questionnaires. The subjects' mean energy intake was calculated on the basis of their basal metabolic rate, taking into account the questionnaire data on daily physical activity at work and leisure time [23].

### Study Design

The study followed a crossover design. Subjects were recruited to participate in a random order on two 1-day studies, separated by approximately one week. The subjects were advised to follow their usual diet during the study period. In addition, they were not allowed to drink alcohol, and were asked to avoid strenuous exercise and sauna for 24 hours before each study day. Moreover, the subjects were advised not to take lipid lowering medication (statins) preceding the study day. The day before the study visit, they were asked to eat an evening meal, in accordance with instructions they had been given, that would provide 15% of the calculated daily energy requirement. The carbohydrate content of the evening meals was 55% energy. The subjects were also asked to fast for 10 to 12 hours after their standardized evening meal, to avoid exercise in the morning of the study, and to arrive at the clinic by car or public transportation.

In the study clinic, height was measured to the nearest 0.1 cm and weight to the nearest 0.1 kg. BMI was calculated as weight (in kg) divided by the square of height (in m). Waist circumference was measured in standing position, with the legs slightly apart, in the midway between the lowest ribs and the iliac crest. Changes of up to two kilos (kg) in weight were allowed during the study. An intravenous cannula was inserted into an antecubital vein in the forearm and an intravenous blood sample (8 ml) was drawn. Thereafter the subjects consumed the test meal within 10 minutes. After the start of the meal intravenous blood samples were collected at 15, 30, 60, 90, 120, 180 and 240 minutes.

Each subject tested two different study meals, a fast-food meal (FF-meal) and a meal, which followed the macronutrient composition of the dietary guidelines [24] and included 52% energy as carbohydrate, 14% energy as protein and 34% energy as fat (REC-meal) on a separate day. The commercial FF-meal consisted of a BigMac hamburger, french fries and light cola. Both test meals contained the same amount of energy (∼3400 kJ = ∼800 kcal). The foodstuffs and the nutrient composition of the study meals are shown in **Table 1**.

**Table 1:** The foodstuffs and the energy nutrient content of the test meals.

REC-meal		g/portion	FF-meal		g/portion
Components	Rye bread^1^	48	Components	Hamburger^2^	210
	Margarine70%^3^	10		French fries^4^	99
	Cheese 5%^5^	37		Light Cola^4^	380
	Cucumber	40			
	Orange juice^6^	260			
	Barley porridge^7^	215			
	Raspberry jam^8^	32			
	Rapeseed oil	15			
	Milk (0% fat)	150			
	Cocoa powder^9^	15			
Total	Carbohydrate	102.4	Total	Carbohydrate	76
	Protein	28.6		Protein	30.7
	Fat	29.8		Fat	39.8
	Total fibre	10.2		Total fibre	7.0
	Energy (kJ)	3359		Energy (kJ)	3326

REC-meal, a meal followed the macronutrient composition of the dietary guidelines; FF-meal, a fast-food meal.

1 Whole-grain rye bread, REAL-ruisleipä; Fazer Ltd, Helsinki, Finland.

2 BigMac hamburger; McDonalds Ltd, Helsinki, Finland.

3 Keiju margarine 70%; Raisio Ltd, Raisio, Finland.

4 McDonalds Ltd, Helsinki, Finland.

5 Aamu cheese 5%; Arla Ltd, Söderkulla, Finland.

6 Orange juice, Vip Appelsiini täysmehu; Vip-Juicemaker Ltd, Kuopio, Finland.

7 Barley porridge prepared with low-fat (1.5% fat) milk.

8 Raspberry jam, Menu Vadelmahillo; Roberts Ltd, Turku, Finland.

9 Dumle cocoa powder; Fazer Ltd, Helsinki, Finland.

### Laboratory analysis

Blood glucose was analyzed by a glucose meter (HemoCue Glucose 201+ meter, LtD, Espoo, Finland) which expresses concentrations as mmol/L of plasma glucose. The HemoCue Glucose system is based on a glucose dehydrogenase method [25]. A quality-control solution recommended by HemoCue was measured twice every study morning; the coefficient of variation (CV) of these measurements was 0.8%. Blood for determination of plasma TG, FFA and insulin were collected in EDTA K2 tubes (Venosafe TM, Terumo Sweden AB, Västra Frölunda, Sweden). Samples were centrifuged for 15 min at 4000 rpm (Rotofix 32, Hettich Zentrifugen, Tuttlingen, Germany) immediately after the sample collecting and the separated plasma was stored at −70°C until analyzed. Plasma concentrations of TG were measured by enzymatic GPO method (Abbott Laboratories, Abbott Park, IL, USA) [26], FFA were measured by enzymatic colorimetric method (NEFA-HR(2),Wako Chemicals GmbH, Neuss, Germany) [27], and insulin was determined by Chemiluminescent Microparticle Immunoassay (CMIA) [28] with Abbott reagents. Fasting total cholesterol at baseline was analyzed by using an enzymatic method (Abbott Laboratories, Abbott Park, IL, USA). All laboratory analyses were carried out at the National Institute for Health and Welfare, Helsinki, Finland using Architect ci8200 analyzer (Abbott Laboratories, Abbott Park, IL, USA).

The inter-assay CV of TG at the levels of 1.35 mmol/L and 4.93 mmol/L were 1.7% and 1.3%, and intra-assay CV was 0.6%. The inter-assay CV of FFA at the levels of 0.59 mmol/L and 1.11 mmol/L were 1.8% and 1.7%, and intra-assay CV varied from 0.3% (low level control) to 0.8 (high level control). The CVs of insulin at the levels of 187 pmol/L and 1191 pmol/L were 1.6% and 2.5%, and intra-assay CV was 1.7%.

### Statistical methods

The 4-h incremental area under the TG, glucose and insulin response curve (IAUC) and the 4-h incremental area over the FFA response curve (IAOC), were calculated using a trapezoidal method [29] for both test meal. In addition, the 2-h glucose and insulin IAUC were also calculated. One subject's insulin IAUC results were excluded from the FF-meal analysis because the fasting insulin value was high and differed markedly from the fasting value of the previous study day and fasting values of other subjects.

The variables with abnormal distribution (glucose and insulin responses) were normalized with logarithmic transformation. An independent sample t-test was used for testing the differences in subjects' baseline characteristics and postprandial responses between study groups. All statistical analyses were done using the PASW Statistics version 18 for Windows® (SPSS Inc., Chicago, IL, USA) [30]; the level of significance was *P*<0.05. The results are expressed as means ± SD.

The sample size calculation for the present study was based on our previous study on elderly men and women [31] and has been calculated for sensitivity of 0.80 and a two-sided significance level of 0.05 indicating 12 subjects sufficient for both study groups.

## Results

### Subjects' characteristics

Baseline characteristics of the subjects are illustrated in **Table 2**. Subjects in the SGI-group were smaller at birth compared with the CON-group. Mean BMI of the study groups were similar (26.9 vs. 26.7 kg/m^2^, *P* = 0.83). In addition, no significant differences were seen between study groups in fasting TG, FFA, glucose nor insulin concentrations. Cholesterol lowering medication (statins) was used by five subjects in the SGI-group and by four in the CON-group. Seven subjects in the SGI-group and five subjects in the CON-group had impaired glucose tolerance (2-h glucose concentration >7.8 mmol/L). Nine subjects of the SGI-group and ten subjects of the CON-group were breastfed. Duration of breastfeeding did not differ significantly between the groups.

**Table 2:** Baseline characteristics of the subjects.^1^

		SGI-group	CON-group	*P*-value^2^
N (male, female)		12 (6,6)	12 (7,5)	
Birth and childhood data	Length of gestation (d)	279.0±9.4	284.3±6.7	0.13
	Birth weight (g)	3129±360	3648±399	0.003
	Birth length (cm)	49.6±2.4	51.3±0.9	0.036
	Birth BMI (kg/m^2^)	12.7±1.0	13.9±1.3	0.019
	Z-score BMI at birth	−0.66±0.85	0.33±1.0	0.019
	Weight at 1 year (kg)	8.9±0.7	10.9±0.6	<0.001
	Length at 1 year (cm)	74.7±3.1	77.1±1.0	0.015
	BMI at 1 year (kg/m^2^)	15.9±0.5	18.3±0.7	<0.001
	Z-score BMI at 1 y	−1.34±0.34	0.46±0.47	<0.001
	Conditional growth^3^	−1.24±0.28	0.40±0.32	<0.001
	Duration of breastfeeding (d)	180±96^4^	172±98^5^	0.86
Adult data	Age (y)	68.0±2.13	68.1±3.23	0.94
	BMI (kg/m^2^)	26.9±2.61	26.7±2.94	0.83
	Waist circumference (cm)	90.1±9.7	92.2±10.2	0.61
	Weight (kg)	75.1±9.0	80.5±9.0	0.16
	Height (cm)	167.1±10.0	173.8±7.5	0.075
	Fasting glucose (mmol/L)	5.32±0.36	5.54±0.51	0.24
	Glucose 2-h (mmol/L)^6^	8.03±1.43	7.91±1.20	0.82
	Fasting insulin (pmol/L)	57.5±21.5	50.6±28.1	0.51
	Fasting cholesterol (mmol/L)	5.06±0.72	4.70±0.85	0.27
	Fasting FFA (mmol/L)	0.46±0.18	0.59±0.18	0.11
	Fasting TG (mmol/L)	1.26±0.22	1.16±0.42	0.47

SGI-group, slow growth during infancy; CON-group, normal growth during infancy; BMI, body mass index; FFA, free fatty acids; TG, triglycerides.

1 Results are mean ± SD.

2 Differences between groups was tested by an independent sample t-test.

3 BMI at age 1 y conditional on BMI at birth.

4 Contain subjects who were breastfed, n = 9.

5 Contain subjects who were breastfed, n = 10.

6 A 2-h oral glucose tolerance test result.

### TG and FFA responses

**Figure 1:**
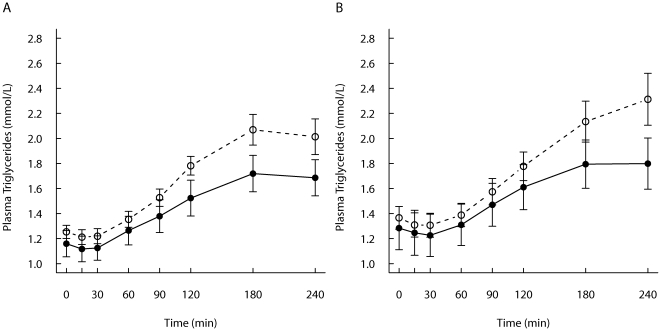
Postprandial triglycerides responses. Mean (± SEM) responses of plasma triglycerides for subjects with slow growth during infancy (open circles, n = 12) and for the control group (filled circles, n = 12) to A) a REC-meal and B) a FF-meal.

**Figure 2:**
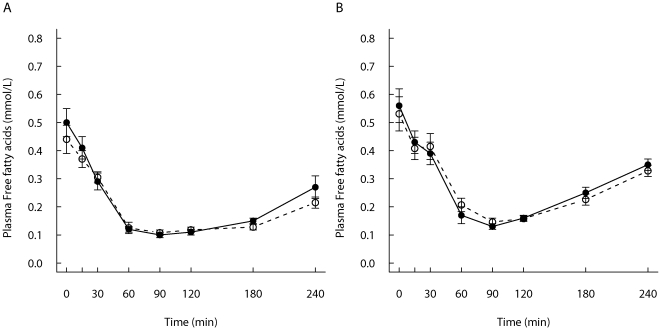
Postprandial free fatty acids responses. Mean (± SEM) responses of plasma free fatty acids for subjects with slow growth during infancy (open circles, n = 12) and for the control group (filled circles, n = 12) to A) a REC-meal and B) a FF-meal.

No significant differences were seen in fasting TG concentrations between the two test meals within the groups. TG concentrations are shown in **Figure 1**, and the postprandial responses expressed as IAUC are shown in **Table 3**. After the study meal, TG concentrations increased and were at the highest level at 3–4 hours after the study meal (**Figure 1**). Early growth affected TG responses, and the 4-h TG responses were higher within the SGI-group compared with the CON-group after the FF-meal (*P* = 0.047) as well as after the REC-meal (*P* = 0.058) (**Table 3**). Early growth did not affect postprandial FFA responses (**Table 3** **,** **Figure 2**). Both study meals suppressed FFA initially, and reached the nadir within 60–90 min. Excluding subjects who used statins in the analysis did not have major impact on the results (data not shown).

**Table 3:** Glucose, insulin, triglycerides and free fatty acids response curve after test meals.^1^

Test meal		SGI-group	CON-group	P-value^2^
REC-meal	Glucose 2-h IAUC (mmol·min/L)	69±56	126±108	0.15
	Glucose 4-h IAUC (mmol·min/L)	76±43	166±140	0.093
	Insulin 2-h IAUC (pmol·min/L)	50492±26993	40406±26566	0.37
	Insulin 4-h IAUC (pmol·min/L)	70843±37064	54206±37104	0.23
	Triglycerides 4-h IAUC (mmol·min/L)	107±41	76±35	0.058
	Free fatty acids 4-h IAOC (mmol·min/L)	64±34	75±31	0.40
FF-meal	Glucose 2-h IAUC (mmol·min/L)	101±64	118±89	0.57
	Glucose 4-h IAUC (mmol·min/L)	113±59	139±109	0.83
	Insulin 2-h IAUC (pmol·min/L)	40319±21790	24925±16241^3^	0.082
	Insulin 4-h IAUC (pmol·min/L)	53458±30357	29849±18618^3^	0.036
	Triglycerides 4-h IAUC (mmol·min/L)	101±51	66±28	0.047
	Free fatty acids 4-h IAOC (mmol·min/L)	67±40	78±47	0.54

SGI-group, slow growth during infancy; CON-group, normal growth during infancy; REC-meal, a meal followed the macronutrient composition of the dietary guidelines; FF-meal, a fast-food meal; IAUC, incremental area under curve; IAOC, incremental area over curve.

1 Results are mean ± SD; n = 12; Glucose and insulin IAUCs were log-transformed prior statistical tests.

2 Differences between groups was tested by an independent sample t-test.

3 n = 11.

### Glucose and insulin responses

**Figure 3:**
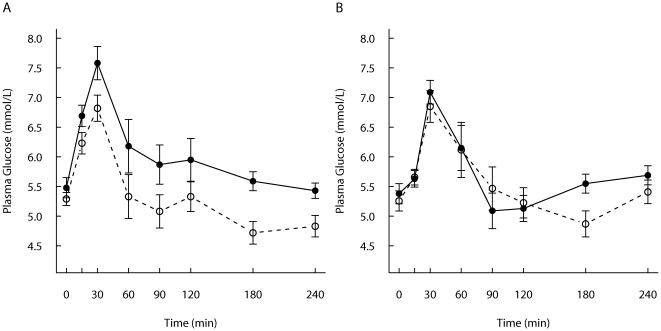
Postprandial glucose responses. Mean (± SEM) responses of plasma glucose for subjects with slow growth during infancy (open circles, n = 12) and for the control group (filled circles, n = 12) to A) a REC-meal and B) a FF-meal.

No significant differences were observed in fasting glucose or insulin concentrations between the study groups. The glucose 2-h and 4-h responses did not differ significantly between the study groups (**Table 3**). Glucose concentrations were peaking 30 min postprandially and approaching baseline values after 60–90 min (**Figure 3**). The glucose concentration after the REC-meal was significantly higher among the CON-group than among the SGI-group at 15–30 and 180–240 min after the ingestion of the meal.

**Figure 4:**
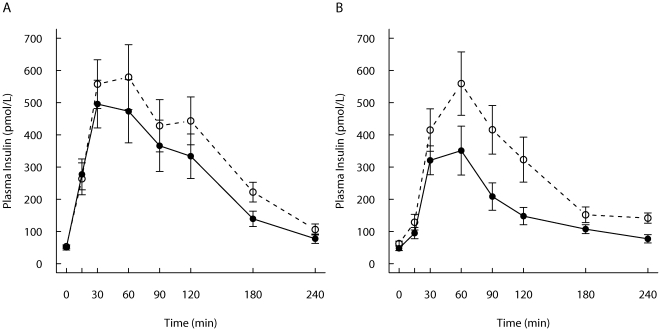
Postprandial insulin responses. Mean (± SEM) responses of plasma insulin for subjects with slow growth during infancy (open circles, n = 12) and for the control group (filled circles, n = 12 except in the FF-meal n = 11) to A) a REC-meal and B) a FF-meal.

The insulin 2-h and 4-h responses were higher for the SGI-group compared with the CON-group (**Table 3** **,** **Figure 4**), however, this difference was (formally) statistically significant only during the FF-meal (*P* = 0.036). Excluding subjects who had impaired glucose tolerance in the analysis did not have major impact on the glucose nor insulin results (data not shown). The 4-h IAUCs ratio of insulin to glucose in the REC-meal differed significantly between the study groups (SGI-group ratio 1066±621 vs. CON-group 466±329; *P* = 0.007). In addition, the ratio of insulin to glucose in the FF-meal were higher in the SGI-group compared with the CON-group (572±369 vs. 318±191; *P* = 0.053).

## Discussion

The objective of this study was to examine the effect of early growth on the postprandial responses of TG, FFA, glucose and insulin in elderly men and women. Our study showed that birth size and growth during infancy have major impact on adult postprandial TG and insulin responses despite similar adult anthropometric characteristics. To our knowledge, this study is the first that have assessed the effect of early growth on postprandial responses in adult life.

In the present study, postprandial TG responses were significantly greater – about 30% higher – for the SGI-group compared with the CON-group. No differences were seen between groups in neither fasting TG concentrations nor fasting FFA nor FFA postprandial responses. Thus, elevated TG responses were not the result of increased adipose tissue lipolysis in the late postprandial state. We suggest that the observed elevated postprandial TG responses might reflect abnormal liver function due to non-optimal liver development during prenatal life and infancy. Liver is a key organ in regulating lipid metabolism, and therefore altered liver growth may permanently alter lipids metabolism. Studies in animal models have demonstrated that undernutrition during gestation affects the liver size at birth [32] and permanently changes lipid metabolism [33] and the microstructure of the liver [34]. Furthermore, it have been shown in epidemiological studies that slow fetal and infant growth [13], [35] or small abdominal circumference at birth, suggesting slow prenatal liver growth, is associated with unfavorable lipid profiles [11].

Two previous studies have found no effect of birth weight on postprandial TG and FFA responses in elderly men and women [15], [16]. Unlike these two studies where the effect of birth weight was investigated, we examined the effect of growth during infancy on postprandial responses. It has previously been observed that fasting TG concentration in adult life is more strongly correlated with slow growth during infancy than with low birth weight [13]. This may partly explain the different findings in the previous studies compared with our findings. However, our study subjects who had grown slowly during the first year of life were also born with a small birth size. Thus, we cannot distinguish between the effect of birth size, infant growth or the combination of the two on TG responses.

Interestingly, whilst glucose responses were slightly, but not statistically significantly, higher in the CON-group in our study, insulin postprandial responses were higher in the SGI-group. A previous study has also shown higher postprandial insulin responses after a standardized breakfast in subjects with low birth weight than in subjects with higher birth weight [17]. Moreover, González-Barranco and co-workers showed that malnutrition during the first year of life had an adverse effect on insulin responses after an OGTT [36]. The higher insulin responses suggest lower insulin sensitivity. In addition to higher insulin responses, insulin resistance may also cause elevated postprandial levels of TGs as observed in our study [37]. Previous studies support this hypothesis of insulin resistance showing that subjects with low birth weight or slow early growth have decreased insulin sensitivity reflecting muscle and adipose tissue insulin resistance [38]–[40]. Both muscle and adipose tissue play a key role in the development of insulin resistance. It is known that those who are thin at birth have reduced lean body mass [41], and have abnormalities in the structure and function of muscle [42], [43]. In addition, undernutrition during gestation may permanently alter the fetal development of adipose tissue and change its metabolic and hormonal functions exposing to the later development of insulin resistance [44], [45]. Excess lipid content in the liver may also cause elevated postprandial lipid responses and insulin resistance [46]. In addition, it has been shown that low birth weight is related to fatty liver disease [47]. Furthermore, as all participants in our study were overweight, the fatty liver, which is closely linked to excess abdominal obesity, may at least partly explain our findings.

It is well known that elevated postprandial lipemia and insulin resistance are closely linked to a number of life style-related diseases, including CVD [18], [19], [48]. A small body size at birth as well as thinness during infancy is also associated with these outcomes [5], [8], [9], [39]. We therefore suggest that our finding of elevated TG and insulin postprandial responses might be one mechanism underlying the increased risk of CVD in people who were small at birth and grew slowly during infancy.

Our study may have some potential limitation. Our study comprised subjects with both normal and impaired glucose tolerance. This might increase the variation of the glucose responses and thus affect the results. We have, however, previously shown that impaired glucose tolerance have effect only on the glucose responses whereas it does not have effect on insulin, TG nor FFA responses [31]. In addition, the limitations of the current work may include a small number of study participants. However, our sample size was based on the power calculations and postprandial studies traditionally include 10 to 30 participants [49]. Our study contained overweight individuals and therefore results may not be directly extrapolated to normal weight individuals. Therefore, our observations need to be confirmed in other studies, which include normal weight subjects as well.

We conclude that small birth size and slow growth during infancy may predict higher postprandial TG and insulin responses in overweight subjects in late adult life. These elevated postprandial responses might be one explanation why people who were small at birth and experiencing slow growth during infancy are at an increased risk of CVD in later life. However, further studies are needed to confirm whether slow growth during infancy without small birth size has effect on postprandial responses.
